# Impact of early protein and energy intakes on neurodevelopment at 2 years of corrected age in very low birth weight infants: A single-center observational study

**DOI:** 10.1371/journal.pone.0218887

**Published:** 2019-06-24

**Authors:** Simon Barreault, Amandine Bellanger, Pauline Berneau, Armelle de La Pintière, Carine Lallemant, Alain Beuchée

**Affiliations:** Department of Pediatrics, University of Rennes 1, Rennes, France; Hopital Robert Debre, FRANCE

## Abstract

**Introduction:**

Aggressive nutritional strategy, particularly enhancing early provision of energy and protein, has appeared to reduce postnatal growth failure and improve later developmental outcomes. But the amount of macronutrients required remains unclear. The aim of this study was to investigate the impact of protein and energy intakes during the first two weeks after birth on neurodevelopmental outcomes.

**Methods:**

This retrospective cohort study of very low birth weight infants born between January 2012 and December 2015 was conducted at one tertiary neonatal intensive care unit. The primary outcome was a neurodevelopmental impairment (NDI) at 2 years corrected age defined by a cerebral palsy or a 24 month Ages and Stages Questionnaires score on any of the five domains lower than 2 standard deviation below the mean score. Multivariable logistic regression analysis was used to adjust for perinatal and postnatal confounders.

**Results:**

Among 245 (73%) infants discharged home alive, 159 (65%) had follow-up at 2 years. Infants with NDI (55/159, 35%) were more likely male gender (67.3% versus 46.2%, P = 0.02) and experienced more patent ductus arteriosus (PDA) ligation (20% versus 5.8%, P = 0.01) than control. After adjusting for confounders, first-week protein intake (OR: 2.27 [CI: 1.07–5.14]; P < 0.05), second-week non-protein energy intake (OR: 1.03 [CI: 1.01–1.05]; P < 0.01) and PDA ligation (OR: 6.81 [1.80–28.46]; P < 0.01) had significant independent association with higher likelihood of NDI at 2 years.

**Conclusion:**

Providing nutrition above the optimal level may not be beneficial and may even be harmful. These results confirm the recent recommendation to decrease amino acid intakes published in the latest ESPGHAN guidelines.

## Introduction

Over the past decade, improvements in perinatal care have resulted in increased survival rates of very low birth weight (VLBW; birth weight <1500 g) infants, having led to a higher incidence of neurodevelopmental sequelae. Recently, a French population-based study of preterm born at 22 to 34 weeks of gestational age (GA) have shown improvements in survival at 2 years’ corrected age (CA) without neuromotor disabilities between 1997 and 2011 but these children have remained at risk of developmental delay [1]. Fetal and postnatal growths are closely associated with neurodevelopmental outcomes for preterm infants. Those who were born small for gestational age (SGA) had lower scores on neurodevelopmental outcomes at different ages than appropriate for GA controls [2]. Greater weight gain early after birth and until one year was associated with better cognitive outcomes at 2 years’ CA [3], especially among VLBW infants. Postnatal growth is compounded when providing adequate nutrient intakes during the first few days is difficult, due to birth contexts and short-term complications of preterm, as patent ductus arteriosus (PDA) or necrotizing enterocolitis (NEC) which interferes with the establishment of an early enteral nutrition [4]. There is considerable evidence that insufficient nutrition in this critical period has long-term negative effects on childhood growth and neurodevelopment [5]. Early nutritional strategy, particularly enhancing early provision of energy and protein, has appeared to reduce postnatal growth failure and improve 18-month developmental outcomes [6–8]. Consequently, the European Society of Paediatric Gastroenterology, Hepatology, and Nutrition (ESPGHAN) and the European Society for Clinical Nutrition and Metabolism regularly proposed targets on parenteral nutrition (PN) for neonates. Initially, 2005's guidelines recommended reaching the goal of 3.5–4.0 g/kg/day of amino acids (AA) and 90–110 kcal/kg/day of energy intakes by the end of the first week [9]. This goal of protein intakes was recently revised downward in 2018's guidelines with 3.5 g/kg/day maximum for all neonates because of a lake of evidence [10]. Quantity of protein and energy required to enable optimal growth and improve neurodevelopment in preterm infants remains a contentious issue.

The aim of the current study was to investigate the impact of protein and energy intakes during the first two weeks after birth on neurodevelopmental outcomes at 2 years’ CA in VLBW infants.

## Methods

### Design

This retrospective cohort study was conducted in a 51-bed tertiary level neonatal intensive care unit (NICU) at Rennes’ University Hospital, France. All inborn and outborn VLBW infants admitted within 24 hours of life from January 2012 to December 2015 and who subsequently had neurodevelopmental assessments at 2 years’ CA in the local perinatal follow-up network were eligible. The infants who had severe congenital brain malformations or chromosomal anomalies, those who had hospital stay less than 7 days or died before follow-up and infants who were lost to follow-up were excluded. The ethics committee of the Rennes University Hospital approved the study. Written informed consent was obtained from the parents of each participating infant before their enrollment in the local perinatal follow-up network. All data were fully anonymized.

### Neurodevelopmental assessment

The primary outcome was a neurodevelopmental impairment (NDI) at 2 years’ CA. Parents reported their child’s development using the second edition of the 24 month Ages and Stages Questionnaire (ASQ) [11] validated in France [12], within time windows between 22 and 26 months. Five developmental domains compose the questionnaire: communication, gross motor, fine motor, problem solving, and personal-social. Each domain contains six items that can be answered with a yes (10 points), sometimes (5 points), or not yet (0 point), resulting in a maximum score of 60 for each domain and an overall maximum ASQ score of 300 points. Children were considered to have a NDI if they have cerebral palsy or a ASQ score lower than 2 standard deviation (SD) below the mean score for the United States reference group on any of the five domains [11].

### Growth assessment

Secondary outcomes were anthropometric measurements performed at birth and 2 years’ CA. Weight, length and head circumference (HC) were recorded at birth and at 2 years’ CA. They were converted to standardized Z-scores according to Olsen curves at birth [13] and to World Health Organization child growth standards at 2 years [14] with the Lambda Mu Sigma method. SGA at birth was defined as a birth weight Z-score below -1.28 SD (i.e. weight for GA below the 10th centile). Z-score changes between the birth and 2 years’ CA (delta Z-score) were calculated as the difference between the 2 years Z-score and the birth Z-score, for each anthropometric measurement.

### Data collection

Baseline clinical data and neonatal comorbidities were retrospectively retrieved from patients' medical records. Nutritional intakes were prospectively collected from our local computerized physician order entry system.

The following maternal details were obtained: singleton or multiple pregnancy, preeclampsia, clinical chorioamnionitis, antenatal steroid therapy initiated (at least two injections), and vaginal or cesarean birth. At birth, baseline clinical data included: gestational age, gender, anthropometric measurements (weight, length, HC) and surfactant replacement therapy.

The presence of comorbidities associated with prematurity were collected such as mechanical ventilation duration, bronchopulmonary dysplasia defined as ventilatory or oxygen requirement at 36 weeks’ PMA, PDA confirmed by echocardiography requiring ibuprofen or surgical ligation, NEC defined as a stage 2A or more according to Bell’s modified classification, and retinopathy of prematurity (ROP) defined as a stage 2 or more according to international classification. Cerebral white matter injuries on magnetic resonance imaging at term for survivors or before death were also reported.

Nutritional intakes data encompassed parenteral and enteral intakes. The daily protein, total energy, non-protein energy and fluid intakes were summed to obtain the median of daily intakes during the first and the second week after birth.

### Nutritional practices

Clinical pathways used in our institution during the study period indicated that parenteral nutrition should be started on the first day of life with 2.4 g/kg/d of AA, 6 g/kg/d of dextrose and 1 g/kg/d of lipids. Subsequent daily increase was 0.5 g/kg/d, 1.2 g/kg/d and 0.5 g/kg/d respectively. Targets for AA intakes were 4 g/kg/d (maximum 4.5 g/kg/d) among infants less than 1000 g at birth or 3.5 g/kg/d (maximum 4 g/kg/d) if birth weight was above 1000g. Target dextrose intake was 15.6 g/kg/d (maximum 18 g/kg/d). Target lipids intake was 3.5 g/kg/d. Early trophic feeding was started within the first day of life, with expressed or pasteurized human milk, for 5 days if the birth weight was less than 1000g or 3 days otherwise. If well tolerated, enteral nutrition was then increased with a daily volume enhancement of 20 to 25 mL/kg/d, PN being replaced gradually. Fortification of maternal or donor human milk was started when enteral intakes reached 80 mL/kg/d. Central line was removed when the infant tolerated 120 mL/kg/d enteral feeding.

### Statistical analysis

Patient’s characteristics are reported as numbers (with percentages) for categorical variables and as medians (with interquartile ranges) for continuous variables. Categorical variables were compared with the Chi square or Fisher’s exact test, and continuous variables were compared with a Mann-Whitney U test, as appropriate. To identify independent predictors of NDI at 2 years’ CA, multivariable logistic regression analysis were performed. In models building, nutritional intakes including protein, non-protein energy and fluid intakes were associated with all clinically relevant variables as GA, birth weight, and neonatal comorbidities. Because birth weight and birth weight Z-score were highly correlated, only birth weight was included in the multivariable models. Optimization for variable selection with stepwise method using the Akaike's Information Criterion (AIC) was applied in Model I. Because GA and birth weight are well known as independent factors of neurodevelopmental outcome and were excluded by variable selection method, Model II kept GA and Model I factors, while Model III kept GA, birth weight and Model I factors. All analyses were performed using the R (R Core Team, 2016) software. Statistical relevance was considered at P value <0.05.

## Results

**Fig 1:**
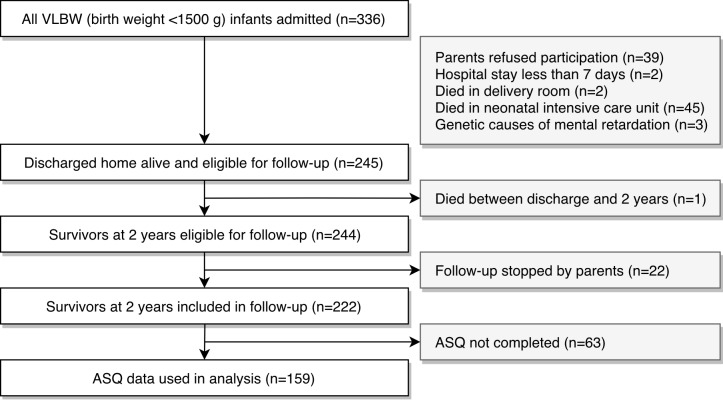
Flowchart. ASQ: Ages and Stages Questionnaire. VLBW: very low birth weight.

Out of a total of 336 VLBW infants assessed, 245 (73%) were discharged home alive and enrolled in the local perinatal follow-up network (Fig 1). Among them, 159 (65%) had results within the time windows of ASQ at 2 years’ CA. 85 (35%) children were lost in follow-up or had not completed ASQ. One child died between hospital discharge and 2 years.

The median GA was 28.4 weeks (26.9 to 29.6 weeks), the median birth weight was 1050 grams (865 to 1240 grams), 55.1% were male, and 13.1% were considered SGA according to their Z-score. The mortality rate during hospital stay was 14%. There was no statistically significant difference between included and excluded infants for the majority of the reviewed variables, including nutrient intakes. There was a longer mechanical ventilation duration (2 days (0 to 12 days) versus 1 day (0 to 6 days), P = 0.006) for excluded compared to included infants.

The characteristics and morbidities of the surviving babies who were assessed at 2 years’ CA were summarized in Table 1, with comparison of the primary outcome. NDI group consisted of 52 infants with ASQ score below threshold, and 3 infants with cerebral palsy. Among them, 8 (14.5%) and 9 (16.4%) had 2 and 3 or more ASQ domains score below threshold, respectively. In the failed domains, 29 (52.7%) children failed in communication area, 18 (32.7%) in gross motor area, 13 (23.6%) in fine motor area, 11 (20%) in problem solving area, and 22 (40%) in personal-social area. Median ASQ scores in NDI group and control were 205 (185 to 220) and 250 (235 to 261), respectively. Infants in NDI group were more likely male gender (67.3% versus 46.2%, P = 0.02) and experienced more PDA ligation (20% versus 5.8%, P = 0.01) than control.

**Table 1:** Baseline characteristics of the follow-up cohort.

	Overall N = 159	NDI group N = 55	Control group N = 104	P value^a^	N
**Baseline clinical data, at birth**					
Multiple pregnancy	51 (32.1)	22 (40)	29 (27.9)	0.17	159
Preeclampsia	44 (27.7)	16 (29.1)	28 (26.9)	0.92	159
Clinical chorioamnionitis	23 (14.5)	10 (18.2)	13 (12.5)	0.46	159
Antenatal steroid therapy	140 (88.1)	48 (87.3)	92 (88.5)	1.00	159
Caesarean	107 (67.3)	39 (70.9)	68 (65.4)	0.60	159
Gestational age (weeks)	28.6 (27.3 ; 29.6)	28.4 (27.3 ; 29.6)	28.6 (27.3 ; 29.6)	0.78	159
Male gender	85 (53.5)	37 (67.3)	48 (46.2)	0.02	159
Weight (grams)	1080 (890 ; 1255)	1110 (903 ; 1310)	1070 (884 ; 1211)	0.36	159
Small for gestational age	19 (11.9)	7 (12.7)	12 (11.5)	1.00	159
Length (cm)	36 (34 ; 38)	37 (35 ; 38)	36 (34 ; 38)	0.30	159
Head circumference (cm)	25 (24 ; 27)	25 (24 ; 27)	26 (24 ; 26)	0.81	155
Surfactant replacement therapy	137 (86.2)	49 (89.1)	88 (84.6)	0.59	151
**Neonatal comorbidities**					
Days of mechanical ventilation	1 (0 ; 6)	0 (0 ; 9)	1 (0 ; 4.25)	0.62	159
Bronchopulmonary dysplasia^b^	53 (33.3)	17 (30.9)	36 (34.6)	0.73	155
Patent ductus arteriosus therapy	43 (27)	14 (25.5)	29 (27.9)	0.89	159
- by ligation	17 (10.7)	11 (20)	6 (5.8)	0.01	158
Necrotizing enterocolitis^c^	13 (8.2)	8 (14.5)	5 (4.8)	0.06	157
Retinopathy of prematurity^d^	20 (12.6)	9 (16.4)	11 (10.6)	0.42	154
White matter injury	18 (11.3)	9 (16.4)	9 (8.7)	0.20	154
**Nutritional intakes, during week 1**					
Protein (g/kg/d)	3.45 (3.06 ; 3.80)	3.53 (3.30 ; 3.83)	3.40 (2.94 ; 3.78)	0.09	157
Non-protein energy (kcal/kg/d)	59 (51 ; 68)	60 (54 ; 68)	58 (50 ; 68)	0.48	157
Total energy (kcal/kg/d)	74 (65 ; 81)	75 (67 ; 83)	72 (64 ; 80)	0.32	157
Fluid volume (ml/kg/d)	130 (110 ; 150)	130 (110 ; 143)	130 (110 ; 150)	0.40	157
**Nutritional intakes, during week 2**					
Protein (g/kg/d)	3.89 (3.53 ; 4.06)	3.88 (3.67 ; 4.05)	3.89 (3.47 ; 4.09)	0.82	157
Non-protein energy (kcal/kg/d)	103 (83 ; 120)	111 (81 ; 124)	101 (83 ; 118)	0.12	157
Total energy (kcal/kg/d)	120 (96 ; 135)	124 (97 ; 139)	117 (98 ; 132)	0.13	157
Fluid volume (ml/kg/d)	160 (150 ; 160)	160 (151 ; 160)	160 (150 ; 160)	0.36	157
**Primary outcome**					
ASQ score below threshold	52 (32.7)	52 (94.5)	-	-	159
Cerebral palsy	3 (1.8)	3 (5.5)	-	-	159
Total ASQ score	235 (210 ; 255)	205 (185 ; 220)	250 (235 ; 261)	-	159

Data are reported as numbers (with percentages) or as medians (with interquartile ranges), as appropriate. ASQ: Ages and Stages Questionnaire. NDI: neurodevelopmental impairment. PMA: postmenstrual age.

^a^ Mann-Whitney U test, Chi square or Fisher’s exact test, as appropriate, alpha = 0.05.

^b^ as ventilatory or oxygen requirement at 36 weeks’ PMA.

^c^ as a stage 2A or more according to Bell’s modified classification.

^d^ as a stage 2 or more according to international classification.

Protein intake increased from 3.45 g/kg per day (3.06 to 3.80) in week 1 to 3.89 g/kg per day (3.53 to 4.06) in week 2. Non-protein energy intake increased from 59 kcal/kg per day (51 to 68) in week 1 to 103 kcal/kg per day (83 to 120) in week 2. No differences were observed between the two groups in the nutritional intakes on univariate analysis.

Multivariable logistic regression analysis were performed to assess the independent contribution of protein and non-protein energy intakes to NDI at 2 years’ CA while controlling for birth weight, GA, gender, fluid intakes and comorbidities associated with neurodevelopmental outcomes (NEC and PDA ligation). In each of our models shown in Table 2, first-week protein intake (odds ratio: 2.27 [CI: 1.07–5.14]; P < 0.05), second-week non-protein energy intake (odds ratio: 1.03 [CI: 1.01–1.05]; P < 0.01), and PDA ligation (odds ratio: 6.81 [1.80–28.46]; P < 0.01) had significant independent associations with higher likelihood of NDI at 2 years. NEC had significant independent association with higher likelihood of NDI only in Model I, while male gender increased the likelihood in Model I and II. GA, birth weight and first-week fluid intake were not significantly associated with NDI at 2 years in our models.

**Table 2:** Multivariable logistic regression analysis of the primary outcome of neurodevelopmental impairment at 2 years' CA.

	Model I		Model II		Model III	
Variables	OR [95% CI]	P value	OR [95% CI]	P value	OR [95% CI]	P value
Gestational age	-	-	1.12 [0.80–1.57]	NS	1.03 [0.72–1.49]	NS
Birth weight	-	-	-	-	1.01 [0.99–1.01]	NS
Male gender	2.21 [1.05–4.76]	<0.05	2.24 [1.06–4.84]	<0.05	1.98 [0.92–4.40]	<0.1
NEC	4.20 [1.01–20.34]	<0.1	4.10 [0.99–19.66]	<0.1	3.77 [0.94–17.53]	<0.1
PDA ligation	6.81 [1.80–28.46]	<0.01	7.44 [1.91–31.85]	<0.01	7.39 [1.89–31.77]	<0.01
Week 1 Protein intake	2.27 [1.07–5.14]	<0.05	2.53 [1.11–6.18]	<0.05	2.78 [1.19–7.04]	<0.05
Week 1 Fluid intake	0.99 [0.97–1.01]	NS	0.99 [0.97–1.01]	NS	0.99 [0.97–1.01]	NS
Week 2 Non-protein energy intake	1.03 [1.01–1.05]	<0.01	1.03 [1.01–1.05]	<0.05	1.02 [1.01–1.05]	<0.05
*AIC*	*181*.*052*		*182*.*623*		*183*.*300*	

AIC: Akaike information criterion. CA: corrected age. CI: confidence interval. NEC: necrotizing enterocolitis. NS: no significant. OR: odds ratio. PDA: patent ductus arteriosus.

Nutritional intakes and all clinical relevant variables were included in models building.

Observations = 149. Missing data: bronchopulmonary dysplasia (n = 4), white matter injury (n = 2), and nutritional intakes (n = 2).

Model I including variables selected with stepwise method using the AIC.

Model II including Model I variables and gestational age.

Model III including Model I variables, gestational age and birth weight.

**Fig 2:**
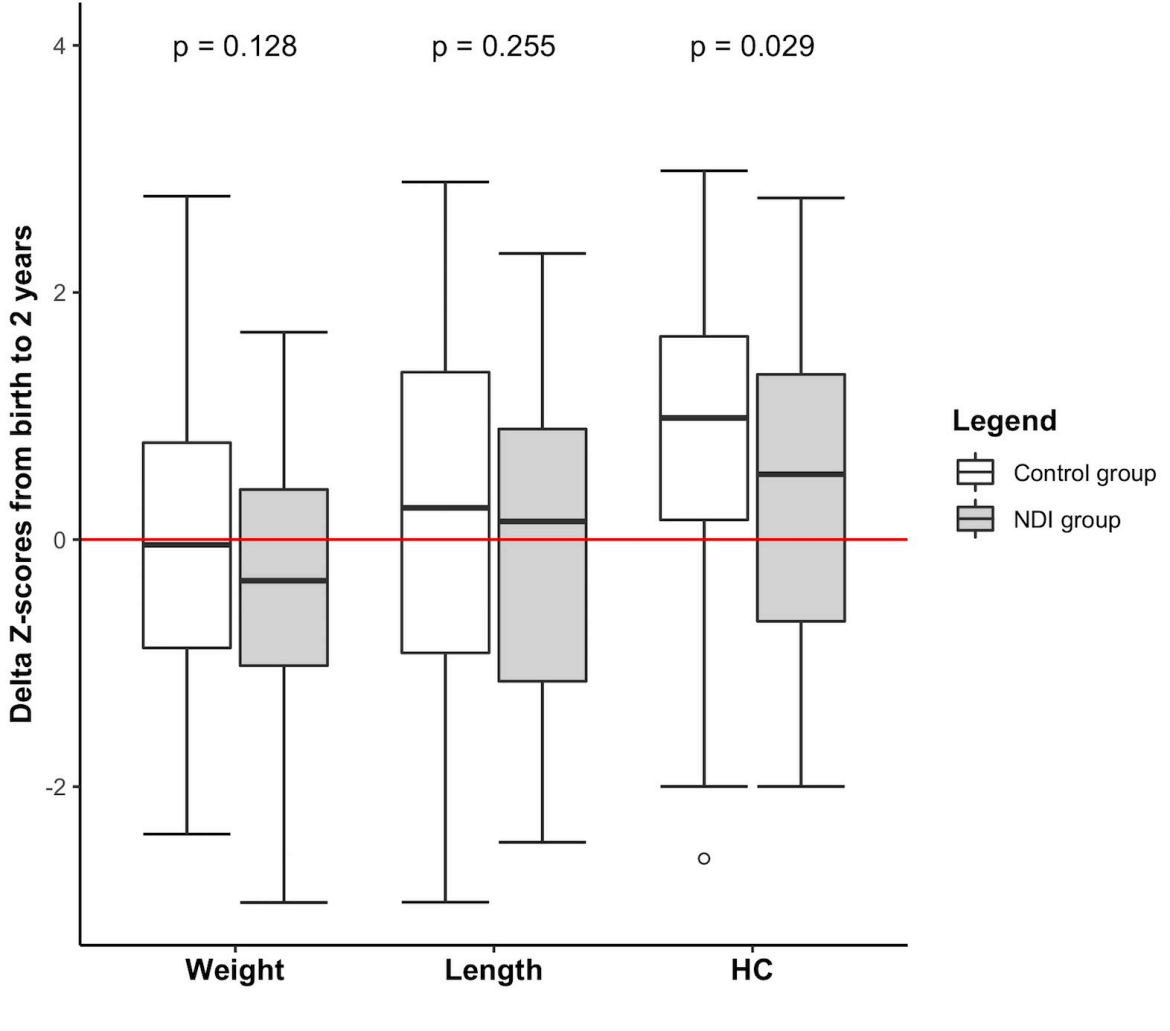
Delta Z-scores between the birth and 2 years’ CA. CA: corrected age. HC: head circumference. NDI: neurodevelopmental impairment. Z-scores at birth are based on Olsen curves [13]. Z-scores at 2 years’ CA are based on World Health Organization child growth standards [14]. Delta Z-score is the difference between the 2 years Z-score and the birth Z-score. Comparison with Mann-Whitney U test, alpha = 0.05.

From birth to 2 years’ CA, growth was similar between groups, except the HC delta Z-score was significantly lower (P = 0.03) in NDI group as compared to control (Fig 2).

## Discussion

The objective of postnatal nutrition in VLBW infants is to achieve a postnatal growth that is similar to fetal growth and coupled with adequate long-term developmental outcomes. The correlation between early provision of protein and later outcomes has been widely documented, while the optimal maximum amount of protein still has to be determined.

In this retrospective cohort study of 245 VLBW survivors, a higher first-week protein intake and a higher second-week non-protein energy intake were associated with higher odds of NDI at 2 years’ CA. Important comorbidities as NEC and PDA ligation were also associated with NDI. These findings contrast with multiple large cohort studies over the past decade that have strongly associated an increase in early protein and energy intake with an improvement of motor and cognitive scores in childhood.

The strengths of this study include the primary outcome assessment method and the adherence to nutritional guidelines recommended over the study period. ASQ was used to assess development delay since is a reliable parent-completed development screening tool which previous studies have indicated high test-retest reliability, inter-observer reliability, and internal consistency [11]. ASQ has shown excellent sensitivity and acceptable specificity for detecting developmental delay in preterm infants at 2 years’ CA compared to Bayley scores [15]. Originally developed with normative data from the United States, the cross-cultural validity of the ASQ was described within several European populations with expecting similar domain scores [16]. Thirty-three percent of children had ASQ scores below threshold, which seem poor compared to previous reports. LIFT cohort [12] revealed 46% of 703 neonates born at less than 35 weeks' GA, but it concerned those born between 2003 and 2006. This difference may be explained by the overall improvement in perinatal care during the last decade. Recently, EPIPAGE-2 cohort [1] showed 42% of 1884 neonates had NDI at 2 years' CA. Unlike this present investigation, all infants were born at less than 31 weeks' GA, not only VLBW infants. Follow-up bias may result in overestimates or underestimates of true rates of impairment. Nevertheless, the follow-up rate of 65% in this study is in line with these surveys, which were 75% and 60% in LIFT and EPIPAGE-2 cohorts, respectively. One limitation of the primary outcome was the inability to record parents' socioeconomic status, which could not be used as explanatory variable, since this is one of the main factors associated with developmental outcomes [1].

The observed negative association between NDI and higher AA intakes supports the recently updated current guidelines that recommend AA intake between 2.5 g/kg per day and 3.5 g/kg per day [10]. Regarding to non-protein energy intakes, previous guidelines suggested 90 kcal/kg per day to prevent a negative nitrogen balance, while the last recommendation is to provide at least 65 kcal/kg per day. Non-protein energy intake in this study was too low to reach an adequate energy to protein ratio. It may be explained by major difficulties to bring sufficient dextrose, which is the major source of non-protein calories in preterm infants, with fluid intake limited by drugs and osmolarity restricted by venous access. The pointed out negative association between non-protein energy intakes and neurodevelopment was misunderstood. One limitation of the present study was that enteral nutrient intakes were not accounted separately. Variations in maternal and donor human milk compositions and variations in nutrients absorptions might have explained some of the effects we observed, more specifically on the second week when the majority of the intakes were enteral. Although neonatal comorbidities were included in logistic regression models, the sample size of the cohort may be insufficient to entirely adjust confounders. Indeed, the major impact of PDA ligation, which is reported here, raises the possibility of fluid restriction leading to a decrease AA intake among these children. Another, PDA ligation may be a surrogate marker for increased illness severity, as "sicker" infants, thus at high risk of NDI, may be more likely to be referred for ligation.

Relatively few studies have investigated the direct effects of neonatal nutritional intake on later neurodevelopment. Stephens et al. [7] were the first to quantify a positive association between first-week average energy and protein intakes and neurodevelopment outcomes in a retrospective analysis of 148 ELBW infants. Every gram per kilogram per day of protein was associated with an 8.2 point increase in Bayley Mental Developmental Index (MDI) at 18 months’ CA. The limitation who was nonexistent at the time of the study was its low amount of protein (1 g/kg/d on day 1 with gradual increase to 2.9 g/kg/d by the end of first week) compared to nutritional intakes which are now recommended. Van den Akker and co-workers [17] found among 132 VLBW infants no difference in growth but a neurodevelopmental advantage at 2 years’ CA for boys that received early 2.4 g/kg/d AA compared to the ones who received only 1.2 g/kg/d AA. However, girls without major disabilities appeared to have a negative association between MDI scores and this early AA administration.

In contrast to above findings, Cester et al. [18] did not find improved outcomes at 2 years’ CA as assessed by Bayley-III scores with a higher protein intake (from 3.3 to 3.8 g/kg/day) before and after changes in nutritional guidelines despite significantly improved early growth and reduced postnatal faltering growth. Blanco et al. [19] reported a lower MDI at 18 months from early increased AA intake (up to 4 g/kg per day, reached as early as 48 hours of life) among ELBW infants, but the difference was no longer significant at the 2-year follow-up. However, the small sample size with several infants lost in follow-up limited the impact of this negative outcome and the study did not used neurodevelopment as the primary outcome.

Overall these studies, evidence appeared to be insufficient to show an effect from increased early protein intake on neurodevelopment at 18 or 24 months’ CA, as confirm the most recent Cochrane systematic review [20]. It is often reported that 2-year outcomes are quite early because many premature infants have impaired neurological development. Beneficial effects of therapies used in the early life may not be manifesting until older ages.

Strong observed associations between neonatal comorbidities, as NEC and PDA ligation, corroborated previous observational studies [21,22] who found these children remained the most likely to develop severe neurodevelopmental disability. Although inadequate total energy intake has been shown to adversely affect head growth and developmental outcomes [23], this present study showed an early higher non-protein energy intake and a smaller head growth among children with NDI. Both energy and protein are necessary to promote net lean tissue accretion and produce normal rates of growth including head growth [24], impacting body composition to adulthood if energy intakes are insufficient during the initial hospital stay [25], with a short leeway since excessive intakes are implicated in promoting overweight and central adiposity [26]. The optimal energy to protein ratio to maximize protein accretion whilst minimizing metabolic harm stay unknown. Furthermore, neonatal comorbidities seem to strongly influence nutritional intakes in early life and their impact was hardly controlled in this study.

In addition to the limitations already described, this investigation has the potential bias inherent in observational uncontrolled study. Outcome data reported here were collected retrospectively and some data, such as the primary outcome, were provided by the parents. Even if nutrient intakes were prospectively collected from our local computerized physician order entry system, it is often reported in nutrition studies that actual intake in 24 hours period can be different from prescription.

## Conclusion

Among a single-center cohort of VLBW infants over a three-year period, a higher early protein intake and non-protein energy intake were associated with higher odds of neurodevelopmental impairment at 2 years’ corrected age. This highlights the contradictory hypothesis that greater protein intakes in early life improve neurodevelopmental outcome like previous studies. However, these results confirm the recent recommendation to decrease amino acid intakes published in the latest ESPGHAN guidelines. When the standard nutrition is below optimal, providing more nutrition may be beneficial. Nevertheless, providing nutrition above the optimal level may not be beneficial and may even be harmful. This study also confirms the strong relationship from neonatal comorbidities to early providing nutrition with an influence on later neurodevelopmental outcome. Further studies are necessary to improve our knowledge and nutritional practices in VLBW infants.

## Supporting information

S1 Dataset Dataset of the cohort. (XLS)
